# No effects of acute stress on monetary delay discounting: A systematic literature review and meta-analysis

**DOI:** 10.1016/j.ynstr.2024.100653

**Published:** 2024-06-03

**Authors:** Paul A.G. Forbes, Jonas P. Nitschke, Nicole Hochmeister, Tobias Kalenscher, Claus Lamm

**Affiliations:** a Comparative Psychology, Institute of Experimental Psychology, Heinrich Heine University Düsseldorf, Germany; b Social, Cognitive, and Affective Neuroscience Unit, Department of Cognition, Emotion, and Methods in Psychology, Faculty of Psychology, University of Vienna, Austria

**Keywords:** Acute stress, Delay discounting, Decision-making, Impulsivity, Systematic review, Meta-analysis

## Abstract

Many everyday decisions, including those concerning our health, finances and the environment, involve choosing between a smaller but imminent reward (e.g., €20 now) and a later but larger reward (e.g., €40 in a month). The extent to which an individual prefers smaller imminent rewards over larger delayed rewards can be measured using delay discounting tasks. Acute stress induces a cascade of biological and psychological responses with potential consequences for how individuals think about the future, process rewards, and make decisions, all of which can impact delay discounting. Several studies have shown that individuals focus more on imminent rewards under stress. These findings have been used to explain why individuals make detrimental choices under acute stress. Yet, the evidence linking acute stress to delay discounting is equivocal. To address this uncertainty, we conducted a meta-analysis of 11 studies (14 effects) to systematically quantify the effects of acute stress on monetary delay discounting. Overall, we find no effect of acute stress on delay discounting, compared to control conditions (SMD = −0.18, 95% CI [-0.57, 0.20], p = 0.32). We also find that neither the gender/sex of the participants, the type of stressor (e.g., physical vs. psychosocial) nor whether monetary decisions were hypothetical or incentivized (i.e. monetary decisions were actually paid out) moderated the impact of acute stress on monetary delay discounting. We argue that establishing the effects of acute stress on the separate processes involved in delay discounting, such as reward valuation and prospection, will help to resolve the inconsistencies in the field.

## 1 Introduction

When we make decisions, we often face a dilemma: do we favour instant gratification over more advantageous long-term benefits? Imagine you receive a bonus from your employer: do you spend the money now or decide to invest it in your pension? The extent to which an individual makes decisions that provide instant gratification (‘smaller-sooner’ rewards) over those which bring greater benefits in the future (‘larger-later’ rewards) can be measured using delay discounting tasks. Delay discounting quantifies how the subjective value of a reward decreases with increasing delay to its receipt. A preference for short-term rewards has been used to explain why people often act against their long-term interests in terms of their health (Bickel et al., 2007; Epstein et al., 2010), financial security (Becker and Mulligan, 1997; Green et al., 1996), and the environment (Berry et al., 2017).

Acute stress is an individual's immediate response to a challenging or aversive situation. It occurs when the homeostasis of an organism is threatened (or perceived to be) by a ‘stressor’, which could be a physical (e.g. pain) or psychological event (e.g. negative social evaluation). The presence of a stressor then triggers a stress response (Chrousos, 2009), resulting in a cascade of biological and psychological changes, which aim to return the organism to homeostasis. This involves various systems, most notably the activation of the fast-acting sympathetic nervous system, resulting in the release of adrenaline and noradrenaline, as well as the slightly delayed response of the hypothalamic–pituitary–adrenal (HPA) axis, resulting in the release of corticosteroids, such as the downstream marker cortisol (De Kloet et al., 2005). This stress response can lead to changes in information processing (for a review: Shields et al., 2016) and decision-making (for a review: Starcke and Brand, 2012) and associated activation differences in the brain regions implicated in these processes, including the prefrontal cortex, anterior cingulate cortex, amygdala, striatum and insula (Pruessner et al., 2004, 2008). However, the specific effect of acute stress on delay discounting—a process involving various mechanisms, including reward processing, prospection, and decision-making—remains unclear. While some studies report increases in delay discounting (e.g., Kimura et al., 2013) others report no effects (e.g., Haushofer et al., 2013). To understand these discrepancies, we conducted a meta-analysis to systematically quantify the effects of acute stress on delay discounting and investigate potential moderators of this relationship.

### 1.1 Delay discounting

**Fig. 1:**
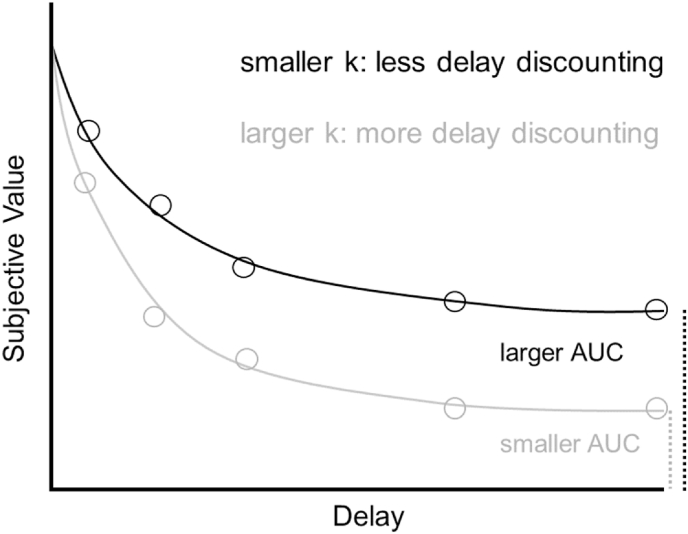
Each circle represents the indifference point at each delay for two hypothetical participants, showing relatively more (grey circles) or less (black circles) delay discounting. The solid lines show the fitted hyperbolic discount functions for the two individuals, with one participant showing a larger k and smaller AUC (i.e. steeper discounting slope; grey line) than the other participant (black line).

In a typical delay discounting task, participants make a series of choices between two options: receiving an amount of money imminently (e.g., smaller-sooner: €20 now) or receiving a larger amount at a later time point in the future (e.g., larger-later: €40 in a month). By varying the delay (e.g., in 3 months, 6 months, 9 months, and 12 months) and the amount of money offered, the indifference points for each delay can be calculated. An indifference point means that the participant is ‘indifferent’ between a smaller-sooner and larger-later reward for a given delay, i.e., they have no preference for either reward and there is a 50% chance of them choosing either option. Both options are assumed to have equal subjective value at the indifference point. For example, when choosing between €20 now and €21 in a month, most people will choose €20 now. Conversely, when choosing between €20 now and €200 in a month, most people will choose €200 in a month (Glimcher, 2011). Hence, individuals will switch from the smaller-sooner to the larger-later option when the value of the larger-later option increases. Determining this switching point allows us to identify the specific value of the larger-later option at which the individual is indifferent between both options. These indifference points can be determined for several different delays (e.g., 1 month, 3 months, 6 months, and so on), quantifying the extent of delay discounting across delays (Glimcher, 2011). A hyperbolic discount function is fitted to the indifference points for each delay (Mazur, 1987):

SV=A/(1+kD)

where *SV* is the subjective value of the discounted value at a given delay, *A* is the reward amount on offer, *D* is the delay, and *k* is the discount rate. Higher values of *k* indicate more delay discounting, i.e., a reduced willingness to choose a larger but delayed reward. Researchers can calculate the area under the curve (AUC) as a measure of delay discounting, with lower values indicating more delay discounting (Fig. 1).

Differences in delay discounting have been associated with a range of behaviours (Reimers et al., 2009). For example, higher rates of delay discounting have been linked to addictive behaviours (Albein-Urios et al., 2014; MacKillop et al., 2011), greater body mass (Jarmolowicz et al., 2014), lower academic performance (Kirby et al., 2005) and financial mismanagement (Hamilton and Potenza, 2012). Thus, understanding if (and how) acute stress influences delay discounting has important implications for educational, health, and financial outcomes. Additionally, multiple ‘state’ effects, such as changes in affect (Wilson and Daly, 2004), can influence delay discounting (for a review: Lempert and Phelps, 2016).

### 1.2 Acute stress and delay discounting

Delay discounting tasks engage multiple neurocognitive processes (Peters and Büchel, 2011), which are potentially vulnerable to the effects of acute stress. Participants must represent the respective values of the smaller-sooner and larger-later rewards and be able to flexibly adjust reward valuation when the delay changes. Acute stress has been implicated in altered reward processing and reward valuation in both behavioural and neuroimaging studies (Forbes et al., 2023; Kumar et al., 2014; Lighthall et al., 2012; Ossewaarde et al., 2011; Tomova et al., 2020). The exact nature and direction of these stress-induced changes remain unclear (Porcelli and Delgado, 2017), with some studies showing a stress-induced blunted valuation of rewards (e.g., Ossewaarde et al., 2011), while other studies suggest enhanced reward salience under stress (e.g., Mather and Lighthall, 2012).

In addition to reward processing, delay discounting also involves cognitive control, in that participants must resolve the decision conflict between two options (Peters and Büchel, 2011). Decision conflict is greatest when the subjective value of the smaller-sooner and larger-later rewards are closer and is related to increased activation in frontal brain regions (Eppinger et al., 2018; McClure et al., 2004). Multiple studies have demonstrated impaired cognitive control processes under acute stress (for a meta-analysis: Shields et al., 2016). However, Plessow et al. (2017) showed that participants under acute stress were able to recruit cognitive control processes when instructed to do so. Moreover, individual differences and task features can determine the extent and nature of the changes in cognitive control under stress (Plieger and Reuter, 2020; Quinn and Shields, 2023).

Finally, delay discounting depends on prospective memory processes, such as thinking about future outcomes. Mentally placing oneself in the future reduces delay discounting and depends on medial temporal brain regions (Patt et al., 2023; Peters and Büchel, 2011). These regions are particularly vulnerable to stress due to a high expression of mineralocorticoid receptors (Koning et al., 2019; Reul and Kloet, 1985). Acute stress disrupts hippocampal-dependent prospection in a virtual navigation task (Brown et al., 2020), and increases in cortisol following a stressor have been linked to disrupted mental simulation of future events (De Wandel et al., 2023).

In sum, delay discounting involves multiple neurocognitive processes, including reward processing, cognitive control, and prospection. These processes are all potentially vulnerable to the effects of acute stress. Examining how and when changes in delay discounting under acute stress could provide insights into which neurocognitive processes are disrupted by acute stress. This could be particularly important for designing interventions to combat the potentially harmful effects of acute stress on delay discounting. For example, there is a strong link between greater delay discounting and addictive behaviours (MacKillop et al., 2011), and stress may be particularly important in further strengthening this link (Fields et al., 2015).

Given the importance of this topic, it has received a great deal of attention through various experimental studies. In a previous meta-analysis, Fields et al. (2014) found a moderate-to-large effect size for the effects of stress on delay discounting in an analysis of 16 studies—greater stress levels were linked to a greater preference for imminent rewards. However, the authors included a wide range of studies in the meta-analysis, and used a broad definition of stress, notably including cross-sectional studies where stress was operationalized by self-reported questionnaire data, such as the Perceived Stress Scale (Cohen et al., 1983), the Life Stress Checklist (Wolfe and Kimerling, 1997), and measures of adverse life experiences (Hamilton et al., 2013; Lovallo et al., 2013). Note, a more recent meta-analysis found a small (r = 0.14) but significant correlation between post-traumatic stress symptoms and delay discounting (Bird et al., 2023). Only a minority of the studies included in Fields et al.’s (2014) meta-analysis experimentally induced acute stress and compared the behaviour of a stress group to a non-stressed control group. Moreover, since Fields et al.’s meta-analysis in 2014, additional experimental studies have been published that induced acute stress and determined the effects of acute stress on delay discounting. However, not all these studies have supported the conclusions of Fields et al.’s (2014). Hence, it is currently not clear whether acute stress impacts delay discounting and how consistent these effects are. More generally assessing the robustness of acute stress' effects on cognition and behaviour is important. For example, in a previous meta-analysis, we found heterogeneous effects of acute stress on social decision-making (Nitschke et al., 2022a, Nitschke et al., 2022b), and a recent replication study failed to find evidence for increased habitual behaviour following acute stress (Smeets et al., 2023). Thus, to systematically quantify the effects of *acute* stress on delay discounting, we conducted a meta-analysis focusing exclusively on studies which experimentally induced acute stress using standardised acute stress protocols (e.g., Kirschbaum et al., 1993). We first examined whether acute stress had an overall effect on delay discounting before investigating several potential moderators of this relationship, given the heterogeneity in behavioural responses to acute stress (Quinn and Shields, 2023).

### 1.3 Moderators

#### 1.3.1 Stressor type

In laboratory studies, both physical and psychological stressors (or a combination of both) can trigger the human stress response, including activation of the HPA-axis. However, the extent and nature of the stress response can vary between different stressors. For example, the Cold Pressor Task (CPT; Hines and Brown, 1936) in which participants must place their hand in very cold water (4 °C or below), has been shown to elicit a less marked stress response, in terms of cortisol and mood ratings, compared to the Trier Social Stress Test (TSST; Kirschbaum et al., 1993). In the latter, participants must give a speech and complete an arithmetic task under social evaluation. Several stress induction protocols have aimed to combine physical and psychological elements, such as the Maastricht Acute Stress Test (MAST; Smeets et al., 2012), Mannheim Multicomponent Stress Test (MMST; Kolotylova et al., 2009), and Socially Evaluated Cold Pressor Task (SECPT; Schwabe et al., 2008). Thus, we aimed to consider the influence of different stressors in delay discounting tasks as different stress-induction methods activate the HPA-axis to a varying degree. Additionally, previous findings show that physical and psychological stressors can interact to influence decision-making (von Dawans et al., 2018).

#### 1.3.2 Gender/sex

Several studies report differences between men and women regarding their psychological and physiological reactions to acute stress (Kudielka and Kirschbaum, 2005). For example, following the TSST, studies have reported that women show higher subjective stress ratings and more negative affect (Kelly et al., 2008; Santl et al., 2019) whereas men show a larger HPA-axis response (Allen et al., 2017; Kirschbaum et al., 1999). Several studies have also reported gender/sex differences in decision-making under acute stress. For example, Lighthall et al. (2009) showed that acute stress can result in more risk-avoidance behaviour in women but more risk-seeking behaviour in men. Gender/sex differences have also been reported in the Iowa Gambling Task following stress induction (van den Bos et al., 2009) and social inference-making abilities (Nitschke et al., 2022). However, no studies (to our knowledge) have specifically investigated gender/sex differences in delay discounting tasks following acute stress.

#### 1.3.3 Hypothetical vs. real rewards

Given the evidence linking stress to differences in reward valuation and sensitivity (Forbes et al., 2023; Kumar et al., 2014; Lighthall et al., 2012; Mather and Lighthall, 2012; Ossewaarde et al., 2011; Porcelli and Delgado, 2017; Tomova et al., 2020), we aimed to determine whether the nature of the reward in the delay discounting task—real vs. hypothetical monetary outcomes—moderated the effect of acute stress. Multiple studies have shown that whether participants are making decisions for ‘real money’ (i.e., participants know that their decisions could actually be paid out) or hypothetical decisions with no monetary consequences makes no difference in delay discounting tasks (Johnson and Bickel, 2002; Lagorio and Madden, 2005; Locey et al., 2011; Madden et al., 2003, 2004). However, the evidence is mixed. Hinvest and Anderson (2010) found that participants showed reduced delay discounting when making real vs. hypothetical reward decisions. Additionally, hypothetical rewards are represented differently in the brain compared to real rewards (Kang et al., 2011; Xu et al., 2018). Thus, we aimed to determine whether the nature of the delay discounting task modulated the effect of acute stress (Vlaev, 2012).

### 1.4 Present study

In the current meta-analysis, we aimed to (1) quantify the effects of acute stress on delay discounting, and (2) investigate whether the participants' gender/sex, the type of stressor, and the nature of the reward (real vs. hypothetical) moderated this relationship. Given that a previous meta-analysis found stress effects on delay discounting (Fields et al., 2014), we hypothesised that acute stress would lead to greater delay discounting–a stronger preference for smaller-sooner over larger later rewards. We compared this to the null hypothesis that acute stress would not have a consistent effect on delay discounting across studies (e.g., Haushofer et al., 2013).

## 2 Method

### 2.1 Study selection

**Fig. 2:**
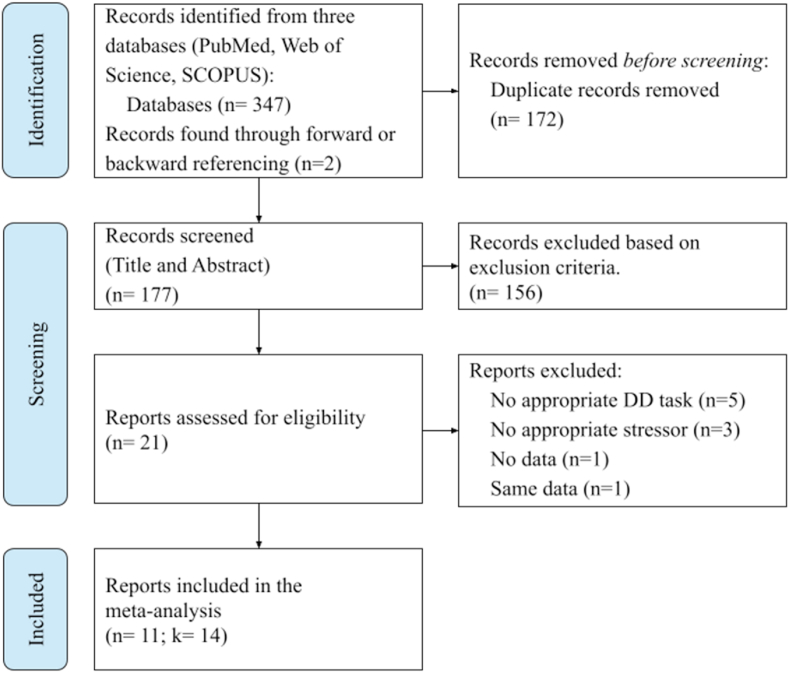
PRISMA flow chart for systematic literature review. Inclusion criteria: (1) Article written in the English language; (2) Article containing original research (i.e., not reviews, opinions, etc); (3) Data had to be based on adult participants (mean age 18 years and above); (4) Study manipulating acute stress, using either of the following tasks: physical stressors, (e.g., Cold Pressor Task (CPT)), psychosocial stressor (e.g., Trier Social Stress Test (TSST), Montreal Imaging Stress Task (MIST)), social evaluative Cold Pressor Task (SECPT), Maastricht Acute Stress Test (MAST)), or variants thereof, with a non-stress control or placebo condition; (5) Study reporting at least one delay discounting task. In addition, studies needed to include measures that could be extracted to calculate an effect size.

We found 347 records in Pubmed (n = 85), Scopus (n = 145), and Web of Science (n = 117) using the following search term: (“Acute Stress” OR “Psychosocial Stress” OR “TSST” OR “cold pressor” OR “CPT” OR “MAST” OR “MIST” OR “social evaluative”) AND (“delay discounting” OR “temporal discounting” OR “discounting” OR “time preference” OR “intertemporal” OR “long-term orientation” OR “self control” OR “time inconsistency” OR “time consistent” OR “dynamic choice inconsistency” OR “reward discounting” OR “choice impulsivity” OR “delayed gratification” OR “delayed reward”). Two additional articles were identified through forward or back referencing. Articles found in more than one database (duplicates = 172) were removed, leaving 177 articles (Fig. 2).

Each article in this list was reviewed independently by three of the authors (PAGF, JPN, NH) to select the articles which met the following criteria: 1) the article was written in English, 2) the article included original data rather than being a meta-analysis, review, opinion, commentary or similar, 3) the data were collected from human research participants (mean age 18 years or above) who did not have a clinical diagnosis of a psychiatric or neurological disorder, 4) the study included an acute stress manipulation (e.g., CPT, TSST, MAST, or variants of these) with a non-stressful control (or ‘placebo’) condition, 5) the study included a task performed up to 2 h after stress induction in which participants made choices between monetary rewards varying explicitly on two dimensions: time to receipt and the magnitude (i.e., smaller-sooner vs. larger-later). Studies in which this information was not explicit but had to be learned through successive decisions were not included (e.g., Byrne et al., 2019). Similarly, studies including self-control elements, such as choices between healthy vs. unhealthy food (e.g., Maier et al., 2015), where the time to the receipt of the reward was not explicitly manipulated, were also not included. Moreover, studies which included the delay discounting task before (Barrios, 1985), during (Flora et al., 2003), or too long after stress induction (i.e., one day after stress induction; Lu et al., 2014) were also excluded. Studies which aimed to induce anxiety (e.g., through threat of shock; Robinson et al., 2015) rather than stress were also not included. Two studies were identified for White et al., 2008, White et al., 2009; however, closer inspection revealed that this concerned the same sample of participants. Thus, we included the data from just one of these studies (White et al., 2008).

We identified 11 studies which met our criteria. Given that three of these studies reported more than one stress effect on delay discounting (Haushofer et al., 2013; Lempert et al., 2012; White et al., 2008), we created a separate entry for each group which left 14 effects in total. Out of these studies, three (Kimura et al., 2013; Lempert et al., 2012; White et al., 2009) were included in the meta-analysis of Fields et al. (2014).

### 2.2 Data extraction and analyses

For all studies we extracted the means, standard deviations and number of participants per condition (e.g. for the stress and control group in between-subject designs) for the key measure of delay discounting reported in the paper. Given the methodological and analytical variations between the studies, the particular measure of delay discounting we extracted differed across papers. Extracted data included discount rates, area under the curve and the proportion or number of immediate (smaller-sooner) rewards chosen (see Table 2). Note that all these measures are correlated as they are derived from the same choice data. Regardless of the measure, we coded the data in such a way that a higher value indicated greater delay discounting–a preference for the smaller sooner reward over the larger later reward. Thus, we reverse-scored AUC values (i.e., 1 – AUC) as higher AUC indicates *less* delay discounting (Lempert et al., 2012; see Table 2). If the data were not reported in the text, supplementary materials, or in an online data repository, we directly contacted the authors and requested the data. If this was not possible, we manually extracted the data from the figures using WebPlotDigitizer (Rohatgi, 2022). If the authors did not respond, or the data could not be extracted from the figures, the article could not be included in the analysis. Effect sizes were calculated using the *escalc* function from the *metafor* package in R (Viechtbauer, 2010). For studies with a between-subjects design, we calculated the standardised mean difference (SMD; Hedges’ g; Hedges and Olkin, 2014). For those with a within-subject design (i.e. a pre-vs. post-stress manipulation), we calculated the standardised mean change score with raw score standardisation (SMCR) due to the correlation between the measurements (Becker, 1988). As the correlations between the pre- and post-measurements are usually not reported, we set *r* to 0.7 which is at the lower end of estimates of the test-retest reliability of delay discounting (between 0.71 and 0.91; Kirby, 2009; Odum, 2011; Simpson and Vuchinich, 2000). Note we repeated the analysis with a more conservative estimate (r = 0.5; e.g., Castrellon et al., 2021) but this did not change the key findings.

To estimate the effect of acute stress on delay discounting, we used a random-effects meta-analysis with restricted maximum likelihood using the *rma.mv* function from the R package *metafor* (Viechtbauer, 2010). We used a multilevel approach to account for multiple effects provided by individual studies (e.g., two effects from Haushofer et al., 2013, Lempert et al., 2012; White et al., 2008). We included a random error term (*study-id*) to represent each study, and this accounted for the multiple effects from some of the studies. Degrees of freedom were adjusted according to Viechtbauer (2010).

Additionally, we ran several moderator analyses. First, we determined whether the gender/sex of the sample moderated the effect of acute stress on delay discounting by comparing the effects from samples of women (n = 3) with those of men (n = 4) and mixed samples (n = 7) of men and women. Next, to determine whether the type of stressor influenced the effect of acute stress, we compared three types of stressors: physical (CPT), psycho-social (TSST, MMST), and combined (including both physical and psycho-social elements: MAST, MMST). We also compared whether the rewards were real (n = 7) or hypothetical (n = 7) and investigated whether this moderated the effect of acute stress on delay discounting.

Where possible, we obtained the mean age of the samples, the gender composition, the stress induction protocol, details concerning the delay discounting task (i.e., reward magnitudes and delays, number of trials, whether decisions were hypothetical or incentivized) and the location of the study (see Table 1, Table 2).

**Table 1:** Overview of the included studies.

Study	Groups	N	Gender	Age	Design	Stressor	Country	Results
Amlung and MacKillop (2014)	Heavy drinkers	84	mixed	22.1 (2.42)	within	TSST (psychosocial)	USA	Stress did not affect delay discounting
Brunelin and Fecteau (2021)	Sham tDCS	14	mixed	28.2 (5.4)	within	MAST (combined)	Canada	Stress reduced preference for immediate reward
Chen et al. (2021)		60 (30 stress)	mixed	20.4 (1.8)	between	CPT (physical)	China	Stress increased preference for immediate rewards
Chipman and Morrison (2015)		63 (29 stress)	women	19.94^a^ (3.95)^a^	between	CPT (physical)	UK	Stress did not affect delay discounting preferences
Haushofer et al. (2013)	early (1) vs. late (2)	142 (71 stress)	men	21.97 (4.23)	between	TSST-G (psychosocial)	Switzerland	Stress did not affect intertemporal choice
Haushofer et al. (2021)		278 (135 stress)	mixed	Not specified	between	TSST (psychosocial)	Kenya	Stress increased the likelihood of early choices
Kimura et al. (2013)		39	mixed	20.80 (2.07)	within	TSST (psychosocial)	Japan	Increased delay discounting for cortisol responders
Krause-Utz et al. (2016)	healthy controls	24	women	27.52 (6.60)	within	MMST (combined)	Germany	Stress did not affect delay discounting
Lempert et al. (2012)	present (1) vs. future oriented (2)	113 (57 stress)	men	20.46 (3.74)	between	Speech (psychosocial)	USA	Trait perceived stress and acute stress interacted to affect delay discounting
Simon et al. (2021)		191 (132 stress)	women	25.00 (3.4)	between	MAST (combined)	Israel	Stress led to an increase in smaller sooner choices
White et al. (2008)	A1+ (1) vs. A1− (2)	71 (35 stress)	mixed	19.29 (1.89)	between	Speech (psychosocial)	Australia	Stress did not affect delay discounting

*Notes*: TSST = Trier Social Stress Test, TSST-G = Trier Social Stress Test for Groups, CPT = Cold Pressor Task, MMST = Mannheim Multicomponent Stress Task, MAST = Maastricht Acute Stress Test. White et al. (2008) used genotyping to identify the presence of the A1 allele of the ANKK1 TaqIA polymorphism and split participants into two groups: A1+ = allele present; A1- = allele absent. Lempert el al. (2012) divided the participants in the stress group into a present-oriented and a future-oriented stress group. Haushofer et al. (2013) gave participants the task either immediately (early) or 20 min after (late) stress induction. Only participants from Brunelin and Fecteau (2021) who received sham (not active) transcranial direct current stimulation (tDCS) were included. The mean age of the participants is reported in years as well as the SD in brackets.

a Data refer to the study's total sample (not the subset used for this analysis).

**Table 2:** Details of the tasks used in the studies.

Study	Task	nt^1^	H^2^	Metric	Amount SS	Delay SS	Amount LL	Delay LL
Amlung and MacKillop (2014)	Delay Discounting	17	yes	Proportion of SS choices	$0.01–$14	immediately	$15	1 week
Brunelin and Fecteau (2021)	Delay Discounting	144	yes	Number of SS choices	Not specified	immediately	Not specified	Not specified
Chen et al. (2021)	Intertemporal Choice	27	no	Proportion of SS choices	¥110 - ¥800	immediately	¥250–¥850	7–186 days
Chipman and Morrison (2015)	Future discounting	3	yes	Preference for SS reward	£400	immediately	£500, £800, or £1200	1 year
Haushofer et al. (2013)	Intertemporal Choice	42	no	Hyperbolic discount parameter (k)	Adapted after each choice starting at 20 CHF	Tomorrow or 6 months	40 CHF	3 months and 1 day to 12 months and 1 day
Haushofer et al. (2021)	Multiple Price List (monetary gains)	48	no	Likelihood of SS choices	400 KES	today or 2 weeks	340 KES–1600 KES	2 weeks or 4 weeks
Kimura et al. (2013)	One-shot delay discounting	1	yes	Delay discounting rate	¥300	immediately	Amount required to justify the delay	1 year
Krause-Utz et al. (2016)	Delay Discounting	40	yes	Delay discounting parameter	randomly selected from Gaussian distribution (M = €20, SD = €10)	immediately	€100	2 weeks or 4 weeks
Lempert et al. (2012)	Delay Discounting	variable	no	Area under the curve (AUC)	started at $2, adapted after each choice (±$0.50)	immediately	$10	1, 2, 30, 180, or 365 days
Simon et al. (2021)	Delay Discounting	60	no	Percentage of SS choices	2 NIS	immediately	20 NIS–200 NIS	1 week–1 year
White et al. (2008)	Two Choice Impulsivity Paradigm	40	yes	Proportion of SS choices	5 points	5 s	15 points	15 s

*Notes*: 1 = Number of trials; 2 = Hypothetical. SS = smaller sooner reward; LL = larger later reward. Brunelin and Fecteau (2021) based their task on Kirby (2009). In all cases, except Lempert et al. (2012), a higher value of the metric indicated more delay discounting (i.e., a preference for the SS reward). Thus, for Lempert et al. (2012), we inverted the AUC values (1 - AUC) so that larger values indicated more delay discounting.

We followed the PRISMA guidelines (Page et al., 2021), and all data and code are available on the Open Science Framework (https://osf.io/uevgk/).

## 3 Results

### 3.1 Study characteristics

We included 11 studies (k = 14 extracted effects) in the analysis, resulting in a sample size of 1097 participants with three studies on women, four studies on men, and seven studies with mixed samples. The studies were published between 2008 and 2021. Sample sizes ranged from 14 to 278, and studies were included from 10 countries (see Table 1). The mean age of the samples ranged from approximately 19 to 28 years old. All the studies reported delay discounting tasks with monetary rewards (or credits which could later be converted to money). In studies which reported delay discounting tasks for other rewards (e.g. vacation days; Chen et al., 2021), we restricted our analysis to monetary rewards to increase the comparability across studies. To induce acute stress, the studies used physical stressors (n = 2; CPT), psychosocial stressors (n = 9, TSST or TSST-G), and stressors combining physical and psychosocial elements (n = 3, MAST or MMST). See Table 1 for further details concerning the included studies.

For each analysis, we report the test statistic and the Q-test for heterogeneity. For the individual effects, we report the SMD and the associated confidence interval (95% CI), test statistic, and p-value. We used Egger's regression test (Egger et al., 1997) to test for publication bias.

### 3.2 Effects of acute stress on delay discounting and potential moderators

**Fig. 3:**
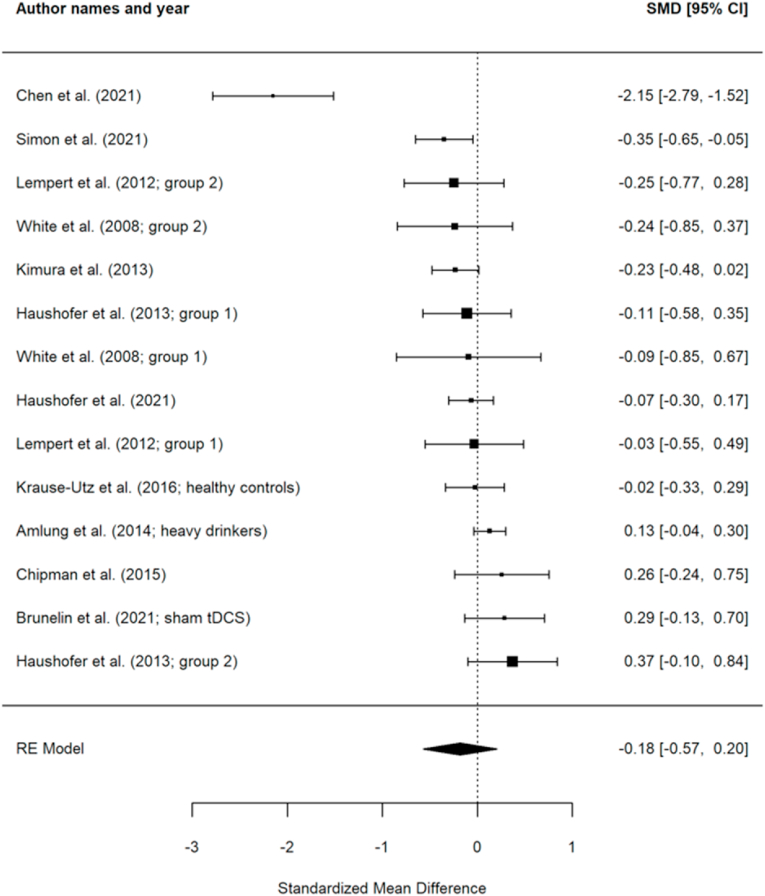
A forest plot showing the effects of acute stress on delay discounting. Negative effects indicate more delay discounting (i.e., a preference for smaller-sooner rewards) under acute stress.

The meta-analysis did not reveal a significant effect of acute stress on delay discounting (SMD = −0.18 (95% CI -0.57, 0.20), SE = 0.17, t (10) = −1.05, p = 0.32). The effects of the individual studies are presented in Fig. 3. There was significant heterogeneity (Q (13) = 60.26, p < 0.01) showing that there was greater heterogeneity amongst the effects than expected from chance alone.

A moderator analysis compared studies with psychosocial stressors (n = 9) to studies with mixed stressors (containing both psychosocial and physical elements; n = 3) to those using purely physical stressors (n = 2) and revealed no significant moderation by stressor type (F (2,11) = 1.62, p = 0.24). There was no significant effect for studies with psychosocial stressors (coded as the reference; k = 9, SMD = −0.06 (95 % CI [-0.55, 0.43]); b = −0.06 (se = 0.22), t (11) = −0.25, p = 0.80), mixed stressors (k = 3; SMD = −0.04 (95 % CI [-0.74, 0.66]); b = 0.02 (se = 0.39), t (11) = 0.05, p = 0.96), or physical stressors (k = 2; SMD = −0.88 (95 % CI [-1.81, 0.05]); b = −0.82 (se = 0.48), t (11) = −1.72, p = 0.11).

A moderator analysis compared studies with women-only samples (n = 3) to men-only (n = 4) samples to mixed-samples of men and women (n = 7) and revealed no significant moderation by gender/sex (F (2,11) = 0.28, p = 0.76). There was no significant effect for women (coded as the reference group; k = 3, SMD = −0.05 (95 % CI [-0.88, 0.78]); b = −0.05 (se = 0.38), t (11) = −0.13, p = 0.90), men (k = 4; SMD = −0.00 (95 % CI [-1.01, 1.00]); b = 0.04 (se = 0.59), t (11) = 0.07, p = 0.94), or for mixed gender/sex samples (k = 7; SMD = −0.32 (95 % CI [-0.91, 0.27]); b = −0.27 (se = 0.46), t (11) = −1.29, p = 0.57).

**Fig. 4:**
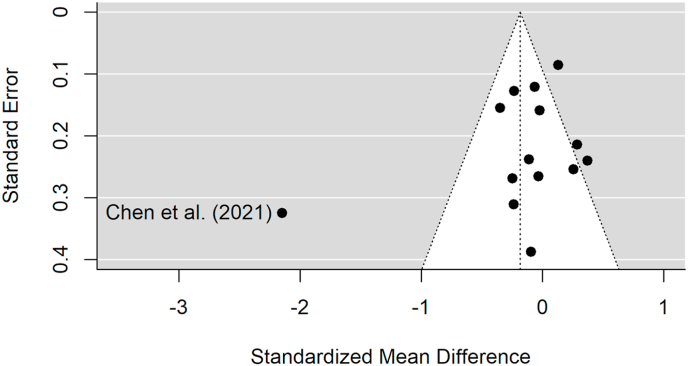
Funnel plot showing the relationship between each study's effects size (SMD) and precision (SE).

We conducted a moderator analysis to compare studies (k = 7) involving hypothetical decisions in the delay discounting task where no actual rewards were paid out to those (k = 7), which incentivized participants' choices by potentially paying out some of the participants' decisions. This revealed no significant moderation (F (1,12) = 1.99, p = 0.18). There was no significant effect for studies with hypothetical decisions (coded as the reference group; k = 7, SMD = 0.04 (95 % CI [-0.46, 0.53]); b = 0.04 (se = 0.23), t (12) = 0.15, p = 0.88) or for those using incentivized decisions (k = 7; SMD = −0.44 (95 % CI [-0.99, 0.10]); b = −0.48 (se = 0.34), t (12) = −1.41, p = 0.18). Egger's test for publication bias revealed no significant bias in the published literature (95 % CI [-0.69, 1.29], p = 0.51). However, the funnel plot (Fig. 4) revealed a visual outlier with the Chen et al. (2021) study. When reanalysing the main effect excluding the Chen et al. (2021) study, the effects of acute stress on delay discounting remained non-significant (SMD = −0.03 [95% CI-0.17, 0.11], SE = 0.06, t [9] = −0.47, p = 0.65).

## 4 Discussion

Humans often prefer short-term rewards at the expense of their long-term goals and interests. Delay discounting tasks measure the extent to which imminent rewards are preferred over delayed but ultimately superior rewards. Acute stress results in a myriad of physiological and psychological changes with potential consequences for decision-making. Previous studies have yielded mixed findings concerning the effects of acute stress on monetary delay discounting. To systematically quantify these effects, we conducted a meta-analysis of 11 studies (k = 14 effects) which experimentally induced acute stress and asked participants to make decisions between ‘smaller-sooner’ and ‘larger-later’ monetary rewards. We did not find a significant effect of acute stress on delay discounting, and moderator analyses revealed that neither the gender/sex of the participants, nor the type of stressor (e.g., physical, psychosocial), nor whether the decisions were hypothetical or incentivized moderated this relationship.

Our findings contrast with a previous meta-analysis that reported a moderate to large effect size between stress and delay discounting (Fields et al., 2014). Fields et al. (2014) included studies using a broader definition of stress, including studies using both acute stress induction paradigms and studies using self-report and retrospective stress measures. Our definition of stress was narrower as we focused our analyses exclusively on studies that experimentally induced acute stress. In addition, we included multiple studies that were published after Fields et al. (2014) and were thus not included in their meta-analysis. Notably, our findings did not reveal any effects of acute stress on delay discounting. Our findings do not exclude the possibility that there are links between chronic measures of stress and delay discounting (Fields et al., 2014). Moreover, a recent meta-analysis found a link between post-traumatic stress symptoms and delay discounting (Bird et al., 2023). However, when linking such chronic or lifetime measures of stress to delay discounting, it is often hard to infer causality and the direction of the effects given that these measures are strongly linked to other factors known to influence both delay discounting and stress levels, such as income and education (Becker and Mulligan, 1997; Jaroni et al., 2004; Shamosh and Gray, 2008). Our findings align with a study showing that 1 h of restraint stress did not affect delay discounting in rats as measured by a preference for larger-later rewards, although it did lead to changes in effort discounting (Shafiei et al., 2012). However, Martinez et al. (2024) found that intermittent social defeat stress led to increased delay discounting (decreased preference for larger-later rewards) but only in rats with low baseline levels of impulsivity and not those with high baseline levels of impulsivity. These findings suggest that the inconsistent effects of stress on delay discounting also generalise to non-human animals and could be dependent on individual differences.

Our moderator analyses did not identify any significant moderators of the effect of acute stress on delay discounting. Neither studies using a purely physical stressor, such as the Cold Pressor Task, nor those using a psychosocial stressor, such as the Trier Social Stress Test, showed an effect of acute stress on delay discounting. Similarly, studies which used a stressor combining both physical and psychosocial stress did not reveal any effects. This suggests that acute stress does not impact delay discounting regardless of the type of stressor. However, given that only two effects used a physical stressor and only three used a combined stressor, a lack of power may have led to these null effects. When we compared samples of only women (n = 3) to those of only men (n = 4) as well as mixed samples of men and women (n = 7), we did not find any effects of acute stress on delay discounting. This suggests that reports of gender/sex differences in behaviour following stress could be restricted to certain domains, for example, during decisions involving risk (Lighthall et al., 2009; van den Bos et al., 2009). Finally, whether the delay discounting task involved real (n = 7) or hypothetical (n = 7) rewards did not moderate the effect of acute stress on delay discounting. We emphasise that the statistical power for the moderated effects was rather low and, as such, should be seen as explorative and interpreted with caution. Based on the current evidence, there does not seem to be a systematic effect that might result in differences in stress-induced delay discounting based on stressor type, gender/sex, or whether monetary choices were incentivized. However, for more conclusive statements, it will be necessary to investigate these effects more systematically using experimental designs that are specific to the research question.

The studies included in the meta-analysis were conducted on healthy participants (the sample from Amlung and MacKillop, 2014 were described as heavy drinkers but without a psychiatric diagnosis). Therefore, it is possible that acute stress changes delay discounting in certain clinical groups, such as in substance use disorders (Fields et al., 2009; Sinha, 2001). Similarly, we only investigated delay discounting of monetary rewards, but other rewards show different patterns of delay discounting (Odum, 2011). People suffering from substance use disorders discount drugs more steeply than money (Coffey et al., 2003; Madden et al., 1997; Odum and Baumann, 2007), and healthy participants discount alcohol and food more steeply than money (Odum and Rainaud, 2003). Thus, investigating how acute stress affects the discounting of non-monetary rewards in clinical populations is an interesting avenue for future work. Additionally, the congruence between the stressor and the reward could also be important. Stress is usually induced in a domain that is not the domain of the delay discounting task, yet it could be that domain-specific effects of stress on delay discounting exist or could be more pronounced. For example, if the source of the stressor, such as pain, is related to the delay discounting task (Tompkins et al., 2016), effects could be stronger. This is an aspect of the analysis which could not be evaluated due to the absence of such studies.

Delay discounting tasks engage multiple neurocognitive processes, such as reward valuation, cognitive control, and prospective memory processes (Peters and Büchel, 2011). The lack of consistent effects on delay discounting could be due to acute stress having opposing influences on these processes. For example, cognitive control processes and prospective memory could be compromised under stress resulting in an enhanced focus on the present, whilst changes in reward sensitivity could reduce the value of the immediate reward (Haushofer et al., 2013). Therefore, understanding how, if, and when acute stress affects the individual processes involved in delay discounting could help to explain our current findings. Furthermore, different physiological aspects of the stress response could have counteracting effects on delay discounting. For example, increasing cortisol levels through hydrocortisone administration enhances preferences for smaller-sooner rewards (Riis-Vestergaard et al., 2018), whereas increases in blood pressure following the administration of yohimbine, which increases noradrenaline action, have been linked to more far-sighted decision making during delay discounting (Herman et al., 2019). Thus, the lack of consistent effects of stress on delay discounting could be due to the countering effects of cortisol and noradrenergic mechanisms. Here the timing of the delay discounting tasks following stress induction could be especially important given the different timescales of these two responses (Joëls et al., 2011). However, it is worth noting that such a time-dependent effect of acute stress on delay discounting was explicitly investigated by Haushofer et al. (2013), who found no effects of acute stress on delay discounting either immediately or 20 min after stress induction. These findings suggest that neither the relatively fast noradrenergic effects of acute stress nor the effects of cortisol, which reaches its peak around 20–30 min post stressor, impact delay discounting. That said, cortisol has both non-genomic and genomic effects on brain function; the latter only manifest after delays longer than 20 min (De Kloet et al., 2005). Hence, we cannot rule out the possibility that the genomic effects of cortisol alter delay discounting if the delay between stress induction and the task is long enough. Until such data exist, however, the most parsimonious explanation for the current findings is that acute stress does not have consistent effects on delay discounting.

It is possible that individual differences could moderate the effects of acute stress. Haushofer et al. (2013) suggested that how people respond to a stressor, for example, as a ‘threat’ as opposed to a ‘challenge’ (Kassam et al., 2009), could determine delay discounting behaviour under stress. Lempert et al. (2012) found that those scoring high on the Perceived Stress Scale (PSS), which measures the extent to which people interpret events in their lives as stressful (Cohen et al., 1983), showed reduced delay discounting under stress. In contrast, those scoring low on the PSS showed enhanced delay discounting when stressed. The importance of individual differences in determining the effects of acute stress on decision-making has recently been demonstrated by Forbes et al. (2023). They showed that the effects of acute stress on effortful prosocial behaviour were moderated by existing prosocial tendencies: participants with more selfish tendencies became more selfish under acute stress, which was not the case for more prosocial individuals (cf. Schulreich et al., 2022). Thus, an exciting avenue for future work would be to determine whether acute stress exacerbates existing differences in impulsivity and impatience in delay discounting tasks (Speer et al., 2022).

We excluded studies which did not involve an explicit choice between a ‘smaller-sooner’ and ‘larger-later’ reward. However, other paradigms have been used to investigate intertemporal decision-making processes under acute stress with mixed results. For example, Byrne et al. (2019) used a task in which participants had to learn the immediate and long-term consequences of their choices based on prior decisions (Otto and Love, 2010). Compared to a control group, stressed participants made more choices which maximised long-term rewards over smaller immediate rewards. Conversely, Lempert et al. (2018) used a willingness-to-wait task to measure participants' persistence to wait for future rewards but found no differences under acute stress. Given the lack of studies implementing these alternative paradigms, it remains to be seen whether acute stress impacts more general intertemporal decision-making processes. Finally, an increasing number of studies have shown that acute stress impacts effort-related decision-making processes (Bogdanov et al., 2021, Forbes et al., 2023, Pavlíčková et al., 2024, Voulgaropoulou et al., 2022). Thus, investigating effort-related decision making, rather than temporal aspects of decision making, could be a fruitful avenue for future work.

## 5 Conclusion

Choosing between short-term rewards and delayed but ultimately more beneficial rewards characterises many everyday decisions. Previous studies have suggested that acute stress—a common everyday occurrence—can exacerbate preferences for inferior short-term rewards. However, not all studies supported this view. In the present meta-analysis, we systematically quantified the effects of acute stress on monetary delay discounting tasks across 11 studies (14 effects): we did not find consistent differences between stressed participants and those in control conditions. Additionally, the gender/sex of the participants, the type of stressor, or whether participants were making real vs. hypothetical decisions did not moderate the relationship between acute stress and delay discounting. Given that acute stress could impact the neurocognitive processes involved in delay discounting, such as reward processing, cognitive control, and prospection, future studies should focus on establishing if or how acute stress affects these individual processes. Also, whether acute stress exacerbates a preference for immediate rewards in certain clinical groups or for non-monetary rewards remains an open question. More generally, our findings highlight the importance of research synthesis approaches for understanding the reliability of acute stress effects on cognition and behaviour.

## Funding

This work was supported by the Austrian Science Fund (FWF, I3381 awarded to CL) and a Marie Skłodowska-Curie Postdoctoral Fellowship from the 10.13039/501100000780European Commission (101107160 awarded to PAGF).

## CRediT authorship contribution statement

**Paul A.G. Forbes:** Writing – review & editing, Writing – original draft, Visualization, Validation, Supervision, Project administration, Methodology, Investigation, Formal analysis, Data curation, Conceptualization. **Jonas P. Nitschke:** Writing – review & editing, Writing – original draft, Visualization, Validation, Supervision, Project administration, Methodology, Investigation, Formal analysis, Data curation, Conceptualization. **Nicole Hochmeister:** Writing – review & editing, Writing – original draft, Visualization, Validation, Methodology, Investigation, Formal analysis, Data curation. **Tobias Kalenscher:** Writing – review & editing, Supervision, Resources. **Claus Lamm:** Writing – review & editing, Supervision, Resources, Project administration, Funding acquisition.

## Declaration of competing interest

None.

## Data Availability

Code and data are available on OSF https://osf.io/uevgk/
